# Assessment of maternal diet inflammatory status and inflammatory markers in human breast milk

**DOI:** 10.1371/journal.pone.0352248

**Published:** 2026-07-02

**Authors:** Courtney B. Slegers, Mark A. Holmes, Caren Biddulph, Judith Maher, Nicolas Roydon Smoll, Melinda M. Dean

**Affiliations:** 1 School of Health, University of the Sunshine Coast, Sippy Downs, Queensland, Australia; 2 Centre for Bioinnovation, University of the Sunshine Coast, Sippy Downs, Queensland, Australia; 3 Sunshine Coast Public Health Unit, Queensland Health, Maroochydore, Queensland, Australia; Nanjing First Hospital, Nanjing Medical University, CHINA

## Abstract

Human breast milk is a complex bioactive fluid containing multi-functional components that support many infant physiological functions. Maternal diet has been demonstrated to influence human milk components; however, how maternal diet impacts inflammatory markers in human milk remains unclear. This study investigated the association between maternal dietary inflammatory status, assessed using the Dietary Inflammatory Index (DII), and the profile of inflammatory markers in breast milk from healthy lactating women, quantified using cytometric bead array. Dietary intake of lactating mothers (n = 101) was assessed using a 24-hr food recall and categorised using the DII as either pro-inflammatory (score > 0) or anti-inflammatory (score < 0). Thirteen inflammatory markers were quantified in breast milk by flow cytometric bead array (13-plex panel: IL-4, IL-2, IP-10, IL-1β, TNF-α, MCP-1, IL-17A, IL-6, IL-10, IFN-γ, IL-12p70, IL-8, free active TGF-β1). All participant diets were categorised as anti-inflammatory diets (DII score range −4.83 to −1.22). Participants’ food intake aligned with dietary guidelines (AUSNUT 2023) for lactating women, with most analysed food parameters classified as anti-inflammatory (19/27). Inflammatory marker analysis revealed a chemokine-dominant profile in breast milk with IP-10, MCP-1 and IL-8 present at the highest concentrations and detected in > 96% of participant human milk samples MCP-1 concentration was weakly associated with DII score (p = 0.025, r^2^ −0.23, Spearman correlation). This study is the first to investigate the inflammatory index of maternal diets in lactating mothers and characterise inflammatory markers in human milk. Further research is required to fully elucidate the relationship between dietary inflammatory status and inflammatory markers in breast milk and their potential impact on infant health, especially of a more diverse cohort.

## Introduction

Human breast milk is a complex bioactive fluid [1] that plays a critical role in reducing infant morbidity and mortality [2]. It contains an abundance of multi-functional components—including fats, proteins, immune components, human milk oligosaccharides (HMOs) [3], growth factors, and micro-organisms—that together support immune development, microbial colonisation, and systemic health [4]. In addition to being nutritionally optimal for infant growth and development [5], breast milk stabilises physiological and circadian rhythms [6], promotes neurological and cognitive development, and helps establish mental health trajectories [7]. Its immune components contribute to innate immunity and prime the adaptive immune system [8]. Among these are cytokines and chemokines: small protein signalling molecules with pro- and/or anti-inflammatory properties essential for immune coordination [9,10]. While cytokines and chemokines have been well characterised in blood serum, our knowledge and understanding of their role in human breast milk remains limited and under-explored.

Maternal diet influences the composition of breast milk, though its impact on inflammatory markers such as cytokines and chemokines remains unclear. These bioactive molecules play key roles in infant immune development [1,2], yet the relationship between maternal dietary patterns and their concentrations in breast milk is not well understood. Other maternal factors such as BMI and nutritional status have also been shown to influence breast milk composition [5,11,12], though it remains unclear whether such factors extend to immune-related molecules. Maternal diet has been shown to influence breast milk composition, including microbiota, lipids, and HMOs [13–15]; however, no studies to date have investigated whether diet modulates concentrations of inflammatory markers in breast milk. The Dietary Inflammatory Index (DII) [16,17] was developed as an evidence-based tool to quantify the inflammatory potential of a person’s diet based on 45 food parameters. Higher scores (> 0) indicate a more pro-inflammatory dietary pattern, while lower scores (< 0) reflect anti-inflammatory pattern. Although the DII has been used to examine maternal dietary associations with breast milk components, such as sodium-potassium (Na + :K+) ratios and fatty acid profiles relevant to infant neurodevelopment [12,18], it has not yet been applied to investigate cytokines or chemokines in human milk.

The relationship between the inflammatory potential of maternal diet and the concentration of inflammatory markers in breast milk remains largely unexplored. This study aimed to investigate whether maternal dietary inflammatory status, assessed using the DII, modulates concentrations of cytokines and chemokines in breast milk. Conducted in a cohort of healthy Australian lactating women, maternal diets were classified as either pro-inflammatory or anti-inflammatory based on DII scores. Inflammatory markers in breast milk were quantified using a bead-based multiplex assay by flow cytometry, and statistical analyses were performed to assess potential associations. Findings from this study aim to advance the understanding of breast milk’s immunological profile and provide preliminary insights into the role of maternal diet in modulating immune-related components in mature human milk.

## Methods

### Study design, ethics and recruitment

This prospective study [19] was conducted in accordance with the National Health and Medical Research Council’s *National Statement on Ethical Conduct in Human Research* and approved by the University of the Sunshine Coast Human Research Ethics Committee (approval numbers: S211573, A242198). A sample of healthy, lactating women (n = 101) from the Sunshine Coast region, Australia, were recruited between 21/05/2021 and 21/05/2022 through convenience sampling. Participation was voluntary, informed consent was obtained in writing, consent was not waived and minors were not included in this study. Participants were screened against inclusion and exclusion criteria as described in a previous study [19]. All participants were three to four months postpartum with established lactation, had delivered their infant at full term (≥37 weeks’ gestation), were English-speaking, and provided voluntary informed consent to participate.

### Sample collection and storage

A single breast milk sample was collected from each participant between 07:00 and 11:00 am, with no milk removal occurring in the two hours prior. Milk was expressed bilaterally using either an electric breast pump or manual expression for 10 minutes, and collected into sterile, single-use polypropylene containers. Samples were immediately placed in the participant’s home freezer (approximately –18°C), and subsequently transferred to – 80°C storage at the research facility [19].

### Maternal dietary analysis and extraction

Participants recorded all food, fluid, and dietary supplement intake consumed in the 24-hours preceding milk collection using the Australian Automated Self-Administered 24-hour (ASA24®) dietary recall tool. Participants were instructed to consume their typical diets and were not following any dietary recommendations during the reporting period. An Accredited Dietitian provided comprehensive education sessions to support accurate self-reporting. Completed submissions were reviewed by an accredited dietitian, and outliers were excluded using predefined, objective cut-off criteria to mitigate over- or under-reporting [19]. Daily mean intakes for 27 dietary components were extracted from the National Institutes of Health ASA24 portal and prepared for DII computation [16,17]. Unit conversions were performed for dietary energy, vitamin B6, caffeine, and n-3 fatty acids to ensure consistency with DII parameters. Alpha-linolenic acid, eicosapentaenoic acid (EPA), docosapentaenoic acid (DPA), and docosahexaenoic acid (DHA) were summed to generate the n-3 fatty acids DII food parameter.

### DII score computation and participant classification

**Fig 1:**
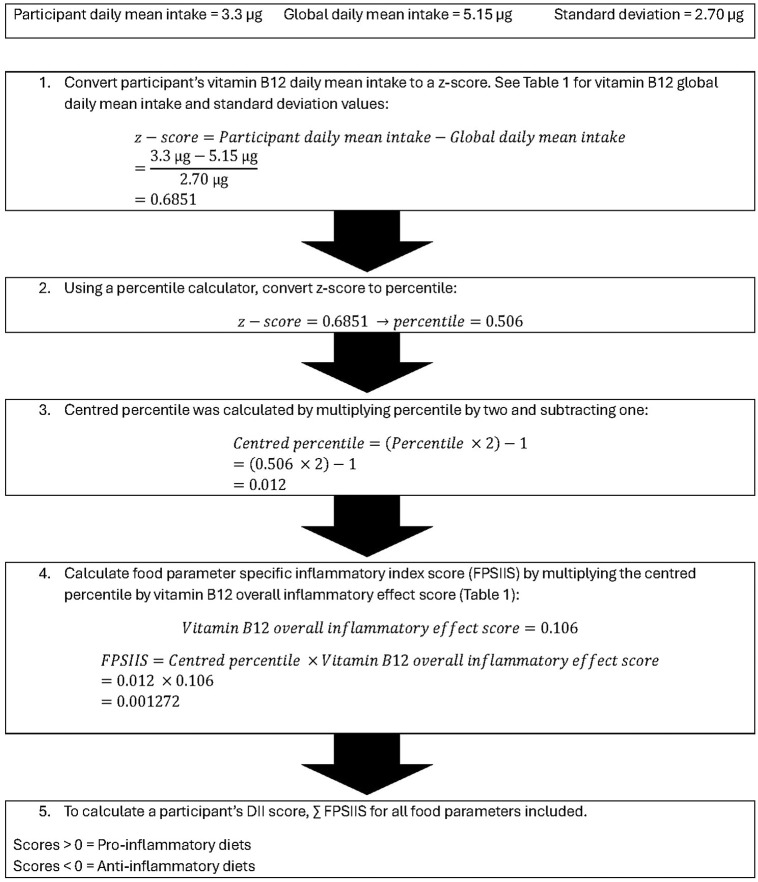
Calculation of Dietary Inflammatory Index (DII) Scores. Step-wise example calculating DII scores using vitamin B12 as a working example [16,17].

Participant dietary inflammatory potential was assessed using the DII, as previously described [16,17] (Fig 1). DII scores were calculated from the 27 ASA24-derived food parameters (Table 1). Participants were classified as having anti-inflammatory diets (DII score < 0) or pro-inflammatory diets (DII score > 0).

**Table 1:** Food parameters extracted from ASA24 for Dietary Inflammatory Index (DII) computation. Adapted from [16,17].

Food Parameter	Overall inflammatory effect score	Global daily mean intake	Standard deviation
Dietary energy (kcal)	0.18	2,056	338
Protein (g)	0.02	79.4	13.9
Carbohydrate (g)	0.10	272.2	40.0
Fibre (g)	−0.66	18.8	4.9
Total fat (g)	0.30	71.4	19.4
Saturated fat (g)	0.37	28.6	8.0
Monounsaturated fatty acid (g)	−0.01	27.0	6.1
Polyunsaturated fatty acid (g)	0.34	13.9	3.8
n-3 fatty acids (g)	−0.44	1.1	1.1
n-6 fatty acids (g)	−0.16	10.8	7.5
Cholesterol (mg)	0.11	279.4	51.2
β-carotene (μg)	−0.58	3,718	1,720
Vitamin A (RE)	−0.40	983.9	518.6
Thiamin (mg)	−0.10	1.7	0.66
Riboflavin (mg)	−0.068	1.7	0.8
Niacin (mg)	−0.246	25.9	11.8
Vitamin B6 (mg)	−0.365	1.5	0.7
Folic acid (μg)	−0.190	273	70.7
Vitamin B12 (μg)	0.106	5.2	2.7
Vitamin C (mg)	−0.424	118.2	43.5
Vitamin E (mg)	−0.419	8.7	1.5
Iron (mg)	0.032	13.4	3.7
Magnesium (mg)	−0.484	310.1	139.4
Selenium (μg)	−0.191	67.0	25.1
Zinc (mg)	−0.313	9.8	2.2
Caffeine (g)	−0.110	8.1	6.7
Alcohol (g)	−0.278	14.0	3.7

### Assessment of inflammatory markers in breast milk using flow cytometric bead array

Breast milk samples were thawed and vortexed before 300 μL was transferred into a soft plastic 500 μL micro-centrifuge tube (Thomas Scientific, USA Cat. #1202K42) and centrifuged (5000 x g, 15 minutes (min), 4°C). The top fat layer was separated from the skimmed breast milk layer by slicing the micro-centrifuge tube with a sharp knife on a cutting board. Skimmed breast milk samples (40 μL) were diluted 1:2 based on a previous study [19]. Dietary assessment was completed on 101 participants; however, breast milk samples were unavailable for 2 participants; therefore, only 99 samples were analysed by flow cytometry.

A LEGENDplex Human Essential Immune Response Panel (13-plex: IL-4, IL-2, IP-10, IL-1β, TNF-α, MCP-1, IL-17A, IL-6, IL-10, IFN-γ, IL-12p70, IL-8, free active TGF-β1; BioLegend Cat. # 740930) was prepared for the flow cytometry protocol as per the manufacturer’s instructions [20]. Inflammatory marker standards (Top standards (STDs)) were reconstituted in 250 µL of assay buffer with gentle mixing (Room Temperature (RT), 10 min) and used to prepare the standard curves via a 6-fold 1/4 serial dilution from Top (C7 (neat) to C1 (1/4096) and assay buffer only (0 pg/mL). Concentrations of Top STD were: TGF-β1 (22,000 pg/mL), IL-17A (13,000 pg/mL), IL-2 (12,000 pg/mL), IP-10 (12,000 pg/mL), IL-1β (11,000 pg/mL), IL-4 (11,000 pg/mL), IL-8 (11,000 pg/mL), IL-10 (10,000 pg/mL), IL-12p70 (10,000 pg/mL), IFN-γ (9,000 pg/mL), IL-6 (9,000 pg/mL), MCP-1 (9,000 pg/mL), TNF-α (9,000 pg/mL). Capture beads (supplied premixed in the kit) were vortexed for 30 secs before use. All standards and samples were run in duplicate. In a V-bottom microplate, 15 µL of each standard or sample were combined with 15 µL of assay buffer and 15 µL mixed beads and incubated (2 hours, RT, plate shaker 800 revolutions per minute (RPM)). Assay plate was then centrifuged (5 min, 250 x g) and supernatant (SN) removed by multichannel pipette without disturbing the bead pellet. To each well, 200 µL of wash buffer was added and plates were incubated for 1 min before centrifuging (5 min, 250 x g) and SN removed. To each well, 15 µL of detection reagent was added and incubated (1 hour, RT, plate shaker, 800 RPM) followed by the addition of 15 µL of streptavidin (SA) phycoerythrin (PE) to each well and a further 30 min incubation (RT, plate shaker 800 RPM). The microplate was centrifuged (5 min, 250 x g) and SN removed, before 200 µL of wash buffer was added to each well and the microplate incubated for 1 min. The microplate was centrifuged (5 min, 250 x g) and SN removed, before 200 µL of wash buffer was added to each well and the plate incubated for 1 min. Next, the microplate was centrifuged and 180 µL of SN removed. Bead pellet was re-suspended in 100 µL of wash buffer.

### Flow cytometry

A three-laser Northern Lights flow cytometer (Cytek Biosciences) was used for inflammatory marker quantification in spectral flow mode according to kit set up instructions. Briefly, beads were first identified based on FSC-A and SSC-B-A. The panel consisted of two bead size populations: Beads A (less FSC/SSC) with six bead populations (IL-1β, IL-2, IL-4, IP-10, TNF-α, and MCP-1) and Beads B (higher FCS/SSC) with seven bead populations (IL-6, IL-8, IL-10, IL-12p70, IL-17A, IFN-γ, free active TGF-β1). Individual beads were identified based on red fluorescence R1 (APC) with detection and median fluorescence intensity determined on B4 (PE). Inflammatory markers were quantified from standard cures using LEGENDplex Data Analysis Software (BioLegend, cloud version).

### Statistical analysis

Participant DII scores were computed using R software (version 4.4.0), incorporating dietary data extracted from the ASA24 portal alongside DII food parameter-specific global daily means, standard deviations, and inflammatory effect scores. Violin plots were generated in R to visualise the cohort’s overall DII distribution, along with anti-inflammatory and pro-inflammatory food parameter-specific DII scores (FPSDIIs).

Descriptive analyses for DII scores and breast milk inflammatory markers were conducted using GraphPad Prism (version 10.4.0). The proportion of participants with detectable concentrations of each inflammatory marker (> 0 pg/mL) was determined using Microsoft Excel (version 2410) and reported as a percentage. Normality testing of DII scores and breast milk inflammatory markers was performed in Prism using the D’Agostino, Pearson, Anderson-Darling, Shapiro-Wilk, and Kolmogorov–Smirnov tests (α ≤ 0.05). DII scores followed a normal distribution, while inflammatory markers were non-normally distributed. Consequently, two-tailed Spearman’s correlations and linear regression analyses were used to evaluate the relationship between DII scores and individual breast milk inflammatory markers.

To explore this relationship further, the cohort was stratified into two subgroups: 1. 25 participants with the highest inflammatory profiles (top 25); 2. 25 participants with the lowest (bottom 25). Participants were sorted on a continuous scale for each marker in the following order: IP-10, MCP-1, IL-8, IL-10, IL-4, TNF-α, IL-6, IFN-γ, IL-12p70, IL-17A, TGF-β1, IL-2, and IL-1β. Spearman’s correlation and linear regression analyses were determined for each subgroup. A p-value of ≤ 0.05 was considered statistically significant for all analyses.

## Results

### Cohort characteristics

Cohort (n = 101) demographics are reported in another study [19]. The majority of participants were between 31 and 40 years of age (65%), ethnically Caucasian (93%), and delivered their first (42%) or second (44%) child. All infants were born healthy at full term with the mean gestation period of 39.4 ± 1.4 weeks.

### Dietary inflammatory profile of lactating mothers

**Fig 2:**
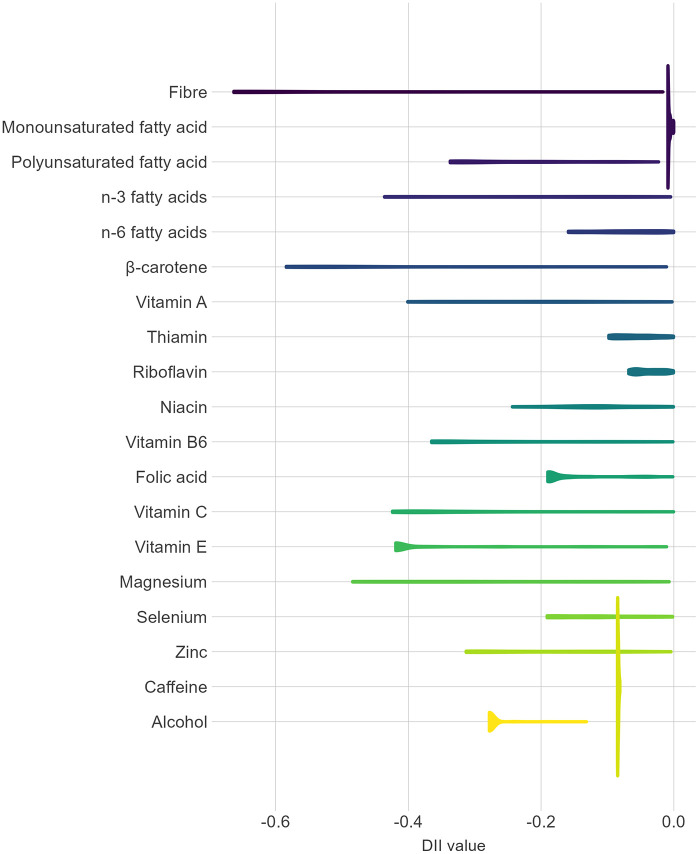
Distribution of anti-inflammatory food parameter contributions to Dietary Inflammatory Index (DII) scores. Range and distribution of food parameter-specific inflammatory index scores (FPSIIS) for 19 anti-inflammatory dietary components (FPSIIS < 0) contributing to the overall Dietary Inflammatory Index (DII) scores in a cohort of healthy Australian lactating women (n = 101). Fibre, β-carotene, and vitamin E exerted the strongest anti-inflammatory influence, reflected in their median FPSIIS. Each plot visualises the density of participant values and central tendency for the respective nutrient.

**Fig 3:**
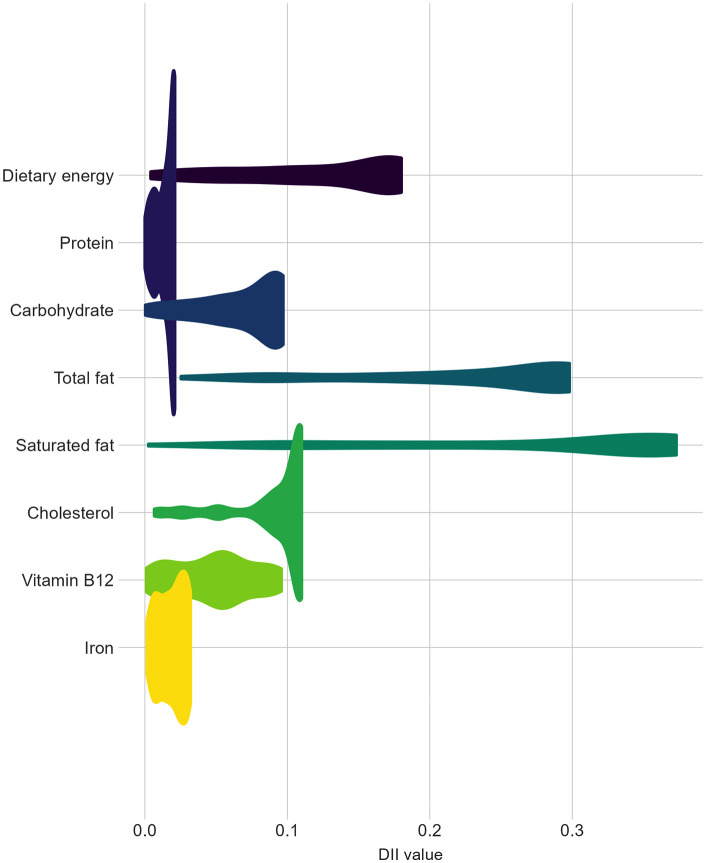
Distribution of pro-inflammatory food parameter contributions to Dietary Inflammatory Index (DII) scores. Distribution of food parameter-specific inflammatory index scores (FPSIIS) for 8 pro-inflammatory dietary components (FPSIIS > 0) contributing to the overall Dietary Inflammatory Index (DII) scores in a cohort of healthy Australian lactating women (n = 101). Total fat and saturated fat had the greatest positive influence on DII scores. The plots represent participant-level variability and central tendency for each nutrient.

**Fig 4:**
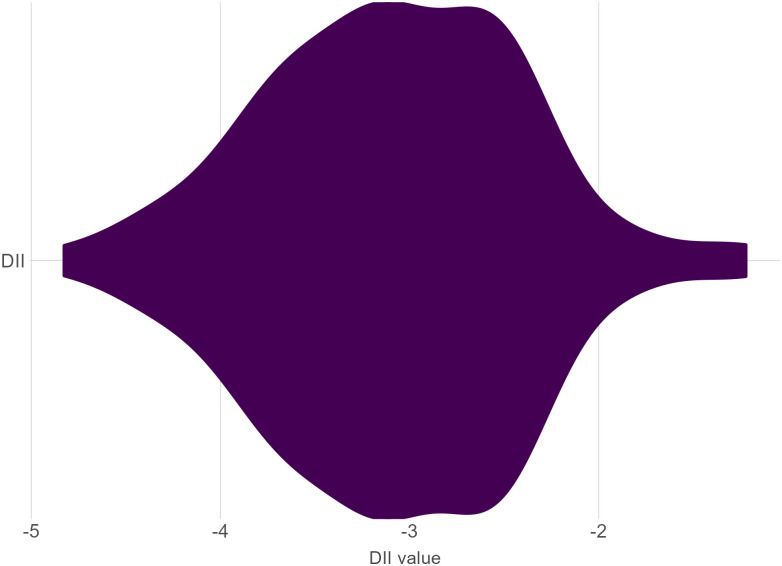
Distribution of overall Dietary Inflammatory Index (DII) scores in lactating women. Distribution of overall Dietary Inflammatory Index (DII) scores for the cohort (n = 101), demonstrating an anti-inflammatory dietary profile across all participants (DII range: −4.83 to −1.22). The distribution approximates normality, with the majority of DII values clustering around −3.0, indicating consistent anti-inflammatory dietary patterns among participants.

Median FPSDIIs ranged from –0.61 to 0.33 (Table 2), with dietary fibre, β-carotene, and vitamin E contributing the strongest anti-inflammatory effect (median FPSDIIs of –0.61, –0.5, and –0.42, respectively). Nineteen of the 27 food parameters had median FPSDIIs < 0 (Fig 2) and eight had pro-inflammatory effects (Fig 3). Participant DII scores ranged from –4.83 to –1.22 (Fig 4), with a median (Q1, Q3) of –3.09 (–3.6, –2.61) (Table 2), indicating that all participants had an overall anti-inflammatory dietary profile.

**Table 2:** Summary of analysed dietary data for a cohort of healthy Australian lactating women (n = 101) compared to global intake and reference values [21].

Food Parameter	FPSIIS Mean (SD)	FPSIIS Median (Q1, Q3)	Cohort Daily Intake Mean (SD)	Global Daily Intake Mean (SD)[41]	n (%) Above Global	Nutrient Reference Values (units/day) [21]
Dietary energy (kcal)	0.12 (0.06)	0.15 (0.08, 0.18)	2,331.46 (642.02)	2,056.00 (388)	71 (70%)	1,949−3,480^*^
Protein (g)	0.02 (0.01)	0.02 (0.01, 0.02)	99.06 (29.02)	79.40 (13.9)	70 (69%)	63 - 67^†^
Carbohydrate (g)	0.07 (0.03)	0.08 (0.05, 0.09)	229.28 (77.55)	272.20 (40)	28 (28%)	–
Fibre (g)	−0.52 (0.18)	−0.61 (−0.66, −0.4)	29.84 (13.89)	18.80 (4.9)	80 (79%)	27 - 30^§^
Total fat (g)	0.23 (0.08)	0.26 (0.18, 0.3)	105.38 (38.6)	71.40 (19.4)	82 (81%)	–
Saturated Fat (g)	0.27 (0.11)	0.33 (0.18, 0.37)	37.85 (15.48)	28.60 (8)	74 (73%)	–
Monounsaturated fatty acid (g)	−0.01 (0)	−0.01 (−0.01, −0.01)	41.73 (17.3)	27.00 (6.1)	81 (80%)	–
Polyunsaturated fatty acid (g)	−0.24 (0.09)	−0.28 (−0.33, −0.18)	17.45 (10.18)	13.88 (3.76)	53 (52%)	–
n-3 fatty acid (g)	−0.23 (0.15)	−0.23 (−0.38, −0.12)	2.39 (1.92)	1.06 (1.06)	81 (80%)	1.4-1.45^¶^
n-6 fatty acids (g)	−0.07 (0.05)	−0.07 (−0.11, −0.03)	14.79 (8.82)	10.80 (7.5)	59 (58%)	10:1^¶^
Cholesterol (mg)	0.09 (0.03)	0.11 (0.09, 0.11)	360.28 (290.87)	279.40 (51.2)	48 (48%)	–
β-carotene (μg)	−0.42 (0.18)	−0.5 (−0.55, −0.3)	3,794.80 (3,938.1)	3,718.00 (1,720)	34 (34%)	–
Vitamin A (RE)	−0.22 (0.12)	−0.21 (−0.32, −0.13)	1,120.74 (800.95)	983.90 (518.6)	44 (44%)	1100^†^
Thiamin (mg)	−0.06 (0.03)	−0.06 (−0.09, −0.03)	1.77 (0.91)	1.70 (0.66)	44 (44%)	1.4^†^
Riboflavin (mg)	−0.04 (0.02)	−0.04 (−0.06, −0.02)	2.20 (0.86)	1.70 (0.79)	75 (74%)	1.6^†^
Niacin (mg)	−0.12 (0.06)	−0.12 (−0.15, −0.07)	25.90 (10.12)	25.90 (11.77)	46 (46%)	17^†^
Vitamin B6 (mg)	−0.26 (0.11)	−0.31 (−0.36, −0.18)	1.63 (0.68)	1.47 (0.74)	57 (56%)	2.0^†^
Folic acid (μg)	−0.16 (0.05)	−0.19 (−0.19, −0.16)	182.76 (156.73)	273.00 (70.7)	25 (25%)	–
Vitamin B12 (μg)	0.05 (0.03)	0.05 (0.03, 0.07)	4.10 (1.92)	5.15 (2.7)	32 (32%)	2.8^†^
Vitamin C (mg)	−0.30 (0.12)	−0.36 (−0.4, −0.24)	90.02 (63.82)	118.20 (43.46)	26 (26%)	80 - 85^†^
Vitamin E (mg)	−0.36 (0.11)	−0.42 (−0.42, −0.37)	16.00 (8.72)	8.73 (1.49)	84 (83%)	11–12^§^
Iron (mg)	0.02 (0.01)	0.02 (0.01, 0.03)	13.63 (5.12)	13.35 (3.71)	47 (47%)	9 - 10^†^
Magnesium (mg)	−0.27 (0.15)	−0.30 (−0.4, −0.15)	436.55 (158.58)	310.10 (139.4)	79 (78%)	310 - 360^†^
Selenium (μg)	−0.12 (0.06)	−0.12 (−0.18, −0.07)	100.04 (57.94)	67.00 (25.1)	76 (75%)	75^†^
Zinc (mg)	−0.20 (0.1)	−0.21 (−0.29, −0.11)	11.89 (4.07)	9.84 (2.19)	64 (63%)	11 - 12^†^
Caffeine (g)	−0.08 (0)	−0.08 (−0.08, −0.08)	0.15 (0.14)	8.05 (6.67)	0 (0%)	–
Alcohol (g)	−0.27 (0.02)	−0.28 (−0.28, −0.28)	2.38 (8.51)	13.98 (3.72)	8 (8%)	0
Overall DII	−3.09 (0.67)	−3.09 (−3.6, −2.61)				

*478−501 kcal/day additional to estimated energy requirement for lactating women based on age, gender, BMR, reference weight and physical activity level; ^†^ Recommended Dietary Intake (RDI); ^‡^ Estimated Average Requirement (EAR); ^§^ Adequate Intake (AI); ^¶^ n-6 fatty acids:n-3 fatty acids

Australian and New Zealand Nutrient Reference Values (NRVs) [21] for lactating women exist for 18 of the 27 food parameters (Table 2). Most cohort nutrient intakes were within the expected NRV. The energy intake of most participants (57.6%) was below the NRV recommendation for lactating women. Alcohol intake exceeded the global mean in 8% of participants, with the NRV recommending abstinence during lactation. Most participants exceeded the global intake average for the 27 food parameters, except caffeine and alcohol (Table 2). No participant consumed more caffeine than the global intake mean, and only eight participants consumed more alcohol.

### Analysis of inflammatory markers in breast milk

Due to insufficient volume in breast milk samples from two participants, inflammatory marker quantification was conducted on 99 participant breast milk samples (n = 99) using flow cytometry. Median concentrations of inflammatory markers ranged from 0 to 1,019 pg/mL (Table 3). Only 5 markers showed a median concentration above 0 pg/mL: IP-10 (1,019 pg/mL), MCP-1 (847 pg/mL), IL-8 (198 pg/mL), IL-10 (53 pg/mL), and IL-4 (34 pg/mL) (Table 3). These five markers were among the most detected, with detection rates exceeding 95% for IP-10, MCP-1, and IL-8. Moderate detection rates were observed for IL-10 (64.6%) and IL-4 (53.5%). TNF-α was detected in 48.5% of participants, with a low median concentration of 0 pg/mL (IQR: 0–26 pg/mL). IL-6, although similarly low in concentration (median 0 pg/mL; IQR: 0–123 pg/mL), was detected in nearly a third of samples (29.3%). In contrast, TGF-β1, IFN-γ, IL-1β, IL-17A, IL-12p70, and IL-2 exhibited both median concentrations of 0 pg/mL and detection rates under 20%, with some markers (IL-17A, IL-12p70, and IL-2) detected in fewer than 10% of participants. These findings highlight substantial variability in immune marker presence across individuals, with only a small subset of markers consistently expressed in the healthy lactating population.

**Table 3:** Descriptive statistics of the concentration of inflammatory markers in breast milk (pg/mL) in a cohort of healthy lactating women (n = 99).

Marker	Median (Q1, Q3)	Mean (SD)	Range	Proportion Detected* (%)
IP-10	1,019 (696, 1,549)	1,291 (1,020)	5,824	96.0
MCP-1	847 (510, 1,524)	1,092 (814)	4,265	97.0
IL-8	198 (139, 356)	304 (294)	1,814	96.0
IL-10	53 (0, 67)	59 (77)	470	64.4
IL-4	34 (0, 121)	90 (165)	1,042	53.5
IL-6	0 (0, 123)	73 (146)	1,001	29.7
TNF-α	0 (0, 26)	30 (72)	521	48.5
TGF-β1	0 (0, 0)	23 (125)	1,167	9.90
IFN-γ	0 (0,0)	40 (131)	1,025	15.8
IL-1β	0 (0, 0)	40 (155)	927	6.90
IL-17A	0 (0, 0)	21 (93)	865	18.8
IL-12p70	0 (0, 0)	30 (95)	728	13.9
IL-2	0 (0, 0)	9 (41)	350	9.90

*Proportion of samples with detectable inflammatory marker measurement where concentration was > 0 pg/mL.

Each participant’s inflammatory marker concentration was analysed against their corresponding DII score using two-tailed Spearman’s correlation and linear regression analysis, with significance defined as p ≤ 0.05. A weak inverse association between DII score and MCP-1 was observed in the 99 participants and found to be significant (r = −0.23, p = 0.0248; Table 4). No other inflammatory marker demonstrated a significant correlation with the DII score.

**Table 4:** Correlation analysis of inflammatory markers in breast milk against overall Dietary Inflammatory Index (DII) scores in a cohort of healthy lactating women (n-99).

Marker	r	95% CI	P-value
IP-10	−0.060	−0.26 to 0.14	0.5538
MCP-1	−0.23	−0.41 to −0.024	0.0248^*^
IL-8	−0.040	−0.24 to 0.16	0.6919
IL-10	−0.18	−0.37 to 0.027	0.0802
IL-4	0.015	−0.19 to 0.22	0.8833
IL-6	−0.11	−0.31 to 0.095	0.2786
TNF-α	−0.024	−0.23 to 0.18	0.8150
TGF-β1	−0.034	−0.24 to 0.17	0.7412
IFN-γ	−0.10	−0.30 to 0.11	0.3241
IL-1β	−0.022	−0.22 to 0.18	0.8326
IL-17A	−0.12	−0.31 to 0.087	0.2459
IL-12p70	−0.10	−0.30 to 0.10	0.3213
IL-2	−0.055	−0.26 to 0.15	0.5864

*Significant finding (p ≤ 0.05).

To further investigate the relationship between breast milk inflammatory marker profiles and DII scores within the cohort, the 25 participants with the highest inflammatory load (top 25) and the 25 with the lowest inflammatory load (bottom 25) were examined. Spearman’s correlation was performed on the four inflammatory markers with the highest median concentrations (IP-10, MCP-1, IL-8, and IL-10) (Table 5). No significant correlations were identified within either subgroup. Correlation plots for IP-10, MCP-1, IL-8, and IL-10 are available in the appendix (S1 Fig).

**Table 5:** Correlation analysis of inflammatory markers in breast milk for 25 samples with the highest mean concentration and 25 samples with the lowest mean concentration against their respective overall Dietary Inflammatory Index (DII) scores.

Marker	Top 25			Bottom 25		
	r	95 % CI	P value	r	95 % CI	P value
IP-10	-0.21	-0.57 to 0.21	0.3047	0.073	-0.34 to 0.46	0.7284
MCP-1	-0.081	-0.47 to 0.34	0.7011	-0.18	-0.55 to 0.24	0.3872
IL-8	0.014	-0.39 to 0.42	0.9476	0.090	-0.33 to 0.48	0.6687
IL-10	-0.077	-0.47 to 0.34	0.7158	-0.38	-0.68 to 0.025	0.0581
IL-4	0.16	-0.26 to 0.53	0.4308	-0.0028	-0.41 to 0.40	0.9895
IL-6	0.096	-0.32 to 0.48	0.6476	-0.039	-0.44 to 0.37	0.8538
TNF-α	-0.016	-0.42 to 0.39	0.9392	-0.046	-0.44 to 0.37	0.8271
TGF-β1	0.31	-0.11 to 0.64	0.1309	NA	NA	NA
IFN-γ	0.27	-0.15 to 0.61	0.1901	-0.25	-0.60 to 0.17	0.2191
IL-1β	0.28	-0.14 to 0.62	0.1703	0.23	-0.20 to 0.58	0.2764
IL-17A	0.27	-0.15 to 0.61	0.1888	0.044	-0.37 to 0.44	0.8341
IL-12p70	0.30	-0.12 to 0.63	0.1408	-0.25	-0.60 to 0.17	0.2191
IL-2	0.17	-0.25 to 0.54	0.4076	0.23	-0.20 to 0.58	0.2764

NA = TGF-β1 bottom 25 values were 0 pg/mL, correlation analysis could not be performed; *Significant finding (p ≤ 0.05).

## Discussion

Understanding the influence of maternal diet on breast milk composition is critical to shaping not only infant nutritional intake but also their immune development, microbiome seeding, and long-term health outcomes. While human milk is well-recognised as a dynamic and immunologically rich fluid, relatively little is known about how maternal dietary patterns, particularly their inflammatory potential, may shape its immunological profile. Prior studies have largely focused on clinical or nutrient-specific models, with few exploring diet-inflammation relationships in populations of healthy lactating women. This study aimed to address that gap by examining the association between maternal DII scores and inflammatory marker expression in human milk. By focusing on a healthy cohort of exclusively breastfeeding mothers at 3–4 months postpartum, this study offers a novel contribution to understanding how dietary patterns may influence milk immunology under non-pathological conditions.

This study recruited a cohort of healthy, lactating Australian women, all of whom demonstrated anti-inflammatory dietary patterns based on their DII scores, which were uniformly <0. These scores reflected diets rich in fibre, vitamin E, and β-carotene – the top three anti-inflammatory food parameters contributing to their overall DII outcome. The breast milk samples collected from this population revealed an overall anti-inflammatory profile with most inflammatory markers present in low concentrations. Among all analytes measured, MCP-1 was the only marker that demonstrated a significant negative association with maternal dietary inflammatory status, suggesting a potential dietary influence on this chemokine. No other inflammatory markers showed significant associations with DII scores.

To our knowledge, this is the first study to examine the relationship between maternal dietary inflammatory potential, measured by the DII, and breast milk inflammatory marker concentrations using a bead-based multiplex assay in a healthy population of breastfeeding women. While previous studies have reported associations between higher DII scores and indirect indicators of breast inflammation such as Na⁺:K⁺ ratio or fatty acid profile, these have not assessed inflammatory cytokine concentrations directly [18]. In the present study, concentrations of all measured markers were low and consistent with reference values reported in the serum of a healthy population [22,23]. Levels of IL-6, IL-8, and MCP-1 were substantially lower than those previously reported in the breast milk of women with mastitis or other inflammatory conditions and fell below typical ranges observed in blood serum from healthy adults [24,25]. These findings offer novel evidence that anti-inflammatory dietary profiles, such as those seen in this cohort, may be associated with an anti-inflammatory breast milk inflammatory marker environment.

Compared to previous studies, our findings highlight a different approach in exploring the link between maternal diet and the immunological profile of breast milk. While existing research has used the DII to examine associations with Na⁺:K⁺ ratios in breast milk, which are indicative of subclinical mastitis and inflammation, such studies were limited by narrow inflammatory marker panels and lacked broader immunological profiling [18]. Others have reported positive correlations between maternal DII scores and milk saturated and monounsaturated fatty acid concentrations, noting potential implications for infant neurodevelopment [26]. In contrast, our study assessed a panel of immune markers directly in breast milk, providing insight into its pro-inflammatory or anti-inflammatory status. The concentrations of inflammatory markers observed in our healthy cohort of lactating women fell below the levels reported in women with mastitis [25] and were consistent with or lower than blood serum concentrations seen in healthy adults [22,23]. This suggests that breast milk, even among healthy women, may maintain a uniquely regulated immunological environment that reflects maternal homeostasis while still prioritising protection for the infant.

Chemokines were the most abundant class of inflammatory markers identified in this cohort’s breast milk samples, with MCP-1 and IL-8 consistently showing the highest concentrations. This chemokine-dominant profile likely reflects the central role of breast milk in supporting neonatal immune development, particularly through chemotactic signalling and immune cell recruitment at mucosal surfaces [4,10]. Rather than indicating pathological inflammation, this profile may represent a functional, and physiologically appropriate immune signal tailored for the infant gut. The observed inverse association between MCP-1 and maternal DII scores suggests that maternal diet may exert a subtle modulatory effect on the inflammatory tone of breast milk, potentially offering downstream protection to the infant. Furthermore, the consistency and detectability of these low-level cytokine patterns in milk support emerging interest [6] in breast milk as a non-invasive biomarker for maternal immune and inflammatory status during lactation. This could have future implications for both maternal monitoring and infant health screening strategies.

This study has several limitations that warrant consideration. The cohort, though valuable for characterising healthy lactating women, did not include a comparison group such as women with pro-inflammatory profiles classified based on DII scores > 0. This restricts the ability to contextualise findings across a broader health spectrum. Additionally, the DII itself may not be the most appropriate tool to determine the inflammatory potential of diets for lactating women as the index was designed for a global population (men and women of all ages 18 + years including all health statuses), whereas this study examined a highly specific cohort (healthy women of childbearing age). Dietary data was collected via the Australian ASA24 tool, which, while validated, relies on self-reporting and is therefore vulnerable to recall bias. The 24-hour dietary recall may not fully represent habitual intake patterns [27]. Furthermore, DII calculation in this study was limited to a subset of dietary components due to data availability, and does not account for maternal nutrient absorption, metabolism, or hormonal influences. Analytically, the study was constrained by the absence of breast milk-specific reference ranges for inflammatory markers, and the relatively narrow distribution of marker concentrations may have limited the detection of meaningful variation. These limitations are compounded by the cross-sectional, exploratory nature of the study, which restricts causal inference and generalisability beyond the studied population. Despite the homogeneous anti-inflammatory findings of the dietary data and inflammatory marker profile of human milk, this cohort could be utilised as a baseline reference group to develop reference ranges or upper limits for inflammatory markers in human milk as study results did not include participants’ complex medical histories or known inflammatory conditions.

To build on these findings, future research should aim to recruit larger and more demographically diverse cohorts of lactating women to capture a broader spectrum of dietary and inflammatory variation. Longitudinal study designs tracking maternal diet and human milk composition across pregnancy and lactation would help clarify temporal relationships and identify critical windows where diet may shape the human milk inflammatory marker profile. Standardisation of milk collection, handling, and analysis protocols, along with the development of human milk-specific reference ranges, is needed to enable valid cross-study comparisons and clinical relevance. Additionally, expanding the exploration of breast milk as a reflective biomatrix for maternal systemic inflammation could offer new avenues for non-invasive postpartum health monitoring and early intervention strategies.

Nevertheless, this study adds to the growing body of evidence showing preliminary insight into how maternal diet may shape inflammatory marker profile of human milk. In a healthy cohort consuming predominantly anti-inflammatory diets, human milk displayed a similarly non-inflammatory immune profile, with chemokines emerging as the predominant marker class. While most markers were not associated with DII scores, a weak inverse correlation between MCP-1 and DII suggests potential for maternal nutrition to influence specific inflammatory components of milk. These findings underscore the importance of exploring breast milk not only as a vehicle for infant immune programming, but also as a non-invasive proxy for maternal inflammatory status.

## Supporting information

S1 Fig Comparison of DII-inflammatory marker correlations between the highest and lowest inflammatory profiles. Comparison of Dietary Inflammatory Index (DII) and inflammatory marker correlation analysis of top 25 and bottom 25 samples based on inflammatory profile stratification of the overall highest median concentration (IP-10, MCP-1, IL-8, and IL-10).
(TIFF)
