# Supplementary figures and images for: Assessment of maternal diet inflammatory status and inflammatory markers in human breast milk
Source: PLoS One. 2026 Jul 2;21(7):e0352248. doi: 10.1371/journal.pone.0352248 (PMC13327152; doi:10.1371/journal.pone.0352248)

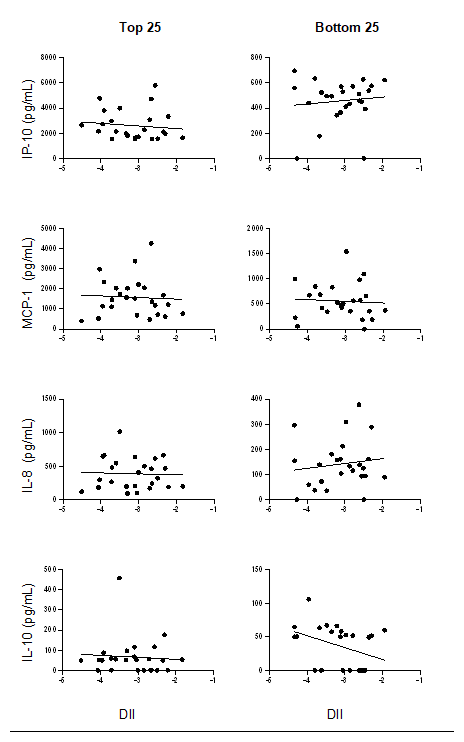

Supplement: S1 Fig — Comparison of Dietary Inflammatory Index (DII) and inflammatory marker correlation analysis of top 25 and bottom 25 samples based on inflammatory profile stratification of the overall highest median concentration (IP-10, MCP-1, IL-8, and IL-10). (TIFF) [file pone.0352248.s001.tiff]
