# Supplementary material for: Assessment of maternal diet inflammatory status and inflammatory markers in human breast milk
Source: PLoS One. 2026 Jul 2;21(7):e0352248. doi: 10.1371/journal.pone.0352248 (PMC13327152; doi:10.1371/journal.pone.0352248)
Supplement: Attachment — Submitted filename: Response to Reviewers.docx [file pone.0352248.s003.docx]

|  | Reviewer #1 Comments to the Author | Author Response |
| --- | --- | --- |
| 1 | Introduction:  - Line 65, there is: “such as BMI and nutritional status”, but to my knowledge have the same meaning; | To avoid potential redundancy and ensure the statement aligns with the cited references, the wording has been revised and ‘nutritional status’ has been replaced with ‘metabolic status’.  *“Other maternal factors such as BMI and metabolic status have also been shown to influence breast milk composition (5,11,12), though it remains unclear whether such factors extend to immune-related molecules.”* |
| 2 | Materials and Methods:  - Lines 109-114, can you better explain the “automated” meaning? The self-administered recall tool is per se criticizable, but without any explanation is maybe worse; | Automated is part of the name of the food recall tool – it is called the ‘Automated Self-Administered 24-Hour Recall’. This food recall tool is a web-based system that participants use to enter their dietary data. Additional detail has been added in the Methods section to better explain the tool, as follows:  *“Participants recorded all food, fluid, and dietary supplement intake consumed in the 24-hours preceding milk collection using the Australian Automated Self-Administered 24-hour (ASA24®) dietary recall tool, a web-based system that participants complete themselves online (i.e. ‘automated’ refers to the digital, self-administered format of the tool) , and uses a structured recall of foods and beverages consumed within a 24-hour period, based on the validated multiple-pass method developed by the National Cancer Institute (20) that has been used for populations such as women of childbearing age and during pregnancy (21,22). Each milk sample was paired with the 24-hour dietary recall corresponding to the period immediately preceding milk collection to align reported dietary intake with the timing of milk sample collection and capture recent maternal dietary exposure at the time of sampling. Participants were instructed to consume their typical diets and were not following any dietary recommendations during the reporting period. Comprehensive education was provided by an accredited practising dietitian to support accurate self-reporting.”* |
| 3 | Results:  - Lines 207-208, there is an explanation? In a western dietary condition, it is surprising | We agree that the anti-inflammatory DII scores were surprising. We have added a sentence under Cohort Characteristics to provide further context to the education background of the study participants, as follows:  *“Overall, this cohort was relatively well-educated, with 95/101 participants having completed a diploma or higher level of tertiary education (1 did not complete Year 12, 5 completed Year 12 only).”* |
| 4 | Results:  - Lines 211-212, there is an explanation? or perhaps can it confirm that the “self administered recall” can give not completed reliable data? | The Automated Self-Administered 24-hour Dietary Recall (ASA24) is a validated, web-based dietary assessment tool based on the multiple-pass method developed by the National Cancer Institute and has been used in populations including women of childbearing age and during pregnancy (20-22).  In this study, a single 24-hour dietary recall was used and intentionally aligned with the timing of milk sample collection to capture short-term maternal dietary intake during milk production, allowing direct comparison between recent dietary exposure and breast milk composition. This approach was selected as not all participants completed multiple recalls, and the paired recall provided the most relevant dietary data for the milk sample analysed. The Methods section was updated as outlined in Response 2 above.  While self-reported dietary assessment is subject to recall bias and measurement error, this limitation is acknowledged in the Discussion and is consistent with standard dietary assessment methods used in lactation and nutrition research. Additional clarification has been added to the Methods to improve transparency around the use and limitations of the ASA24 tool (see Response 2). |
| 5 | Discussion:  - Lines 295-300, it can be understandable that anti-inflammatory diets do not stimulate pro-inflammatory cytokines, but can you explain better the MCP-1 behavior? Besides what is showed in Lines 328-334? | The following wording has been added to clarify the biological role of MCP-1 and to note that MCP-1 concentrations in human milk have been reported to vary across studies and lactation stages:  *“MCP-1 (also called CCL2) is a chemokine that recruits monocytes to site of inflammation, where they can subsequently differentiate into macrophages or dendritic cells (10 Turner et al). MCP-1 concentrations in human milk have been reported to vary across studies and lactation stages, with some studies observing higher concentrations in early lactation and others reporting increased levels during inflammatory conditions such as mastitis (30,31). This chemokine-dominant profile likely reflects the central role of breast milk in supporting neonatal immune development, particularly through chemotactic signalling and immune cell recruitment at mucosal surfaces (4,10).”*  We do not have any further explanation for the modulation of this chemokine, but as our understanding of the role of inflammatory markers improves, further research resulting from the publication of this study and the detailed methods for quantification may identify specific mechanisms. |
| 6 | Discussion:  - Lines 341-343, this is of course a limitation, but I consider much more important what I comment before of the recall tool as the authors suggest in lines 348-350; | The use of a single 24-hour dietary recall and its limitations are acknowledged in the Discussion. As outlined in response to Reviewer 1, Comments 2 and 4, the ASA24 is a validated dietary assessment tool based on the multiple-pass method and has been used in populations including women of childbearing age and during pregnancy (20-22).  In this study, the 24-hour dietary recall was aligned with the timing of milk sample collection to ensure consistency between reported intake and the sampled milk. While a single recall does not fully represent habitual intake, participants were instructed to maintain their usual dietary patterns and the recall captured intake from a typical day without dietary modification.  We have updated the discussion to include the following:  *“Dietary data was collected via the Australian ASA24 tool, which, while validated (21,22), relies on self-reporting and is therefore subject to recall bias and day-to-day variation in dietary intake. The ASA24 relies on participant recall and requires time to complete and a basic level of computer literacy. Dietary data were reviewed by an accredited practising dietitian to ensure completeness and plausibility, with predefined cut-offs applied to identify potential under- and over-reporting (<500 or >3500 kcal/day) (32). The 24-hour dietary recall may not fully represent habitual intake patterns (33). Additionally, a single morning milk sample was collected to standardise sampling conditions, minimise participant burden, and avoid disruption to infant feeding. As this study did not aim to assess diurnal variation, a single time-point sample was considered appropriate to meet the study objectives (34).”* |
| 7 | Discussion:  - Lines 350-352, it is not feasible in similar conditions; | This statement refers to the inherent limitations of the DII calculation rather than feasibility. While many dietary parameters were included, the DII score was calculated using the components available within the ASA24 dataset.  We are unsure what feasibility refers to from the reviewer. We have adjusted this sentence (see below) to reflect the food parameters included. Refer also to previous responses and further explanation provided on the ASA24.  *“Furthermore, the DII calculation in this study was limited to a subset of dietary components due to data availability, and does not account for maternal nutrient absorption, metabolism, or hormonal influences. Analytically, the study was constrained by the absence of breast milk-specific reference ranges for inflammatory markers, and the relatively narrow distribution of marker concentrations may have limited the detection of meaningful variation.”* |
| 8 | Discussion:  - Lines 376-378, please explain better. | As outlined above, further information regarding MCP-1 was added earlier in the Discussion, and we do not have a mechanism at this stage.  This sentence in the final part of the Discussion has been clarified (see below) to better explain the potential biological relevance of the MCP-1 association. We have been careful not to overstate or overinterpret these results in an evolving field of research, and this publication opens many avenues for further exploration.  *“While most inflammatory markers were not associated with DII scores, a weak inverse correlation between MCP-1 and DII suggests the inflammatory status of a lactating mother’s diet may influence MCP-1 concentrations in human milk, suggesting a potential effect on chemokines. These findings underscore the importance of exploring breast milk not only as a vehicle for infant immune programming, but also as a non-invasive proxy for maternal inflammatory status.”* |

|  | Reviewer #2 Comments to Author | Author Response |
| --- | --- | --- |
| 1 | Considering the aim of the study: 'This study aimed to investigate whether maternal dietary inflammatory status, assessed using the DII, modulates concentrations of cytokines and chemokines in breast milk' and the fact that these relationships were confirmed only for MCP-1, and that the study indicated the need for further research, I believe the title should be modified to state that these are pilot studies. This is also confirmed by the numerous limitations of the study. | This study was not designed as a pilot study, and the sample size was considered appropriate for the exploratory observational design. While only MCP-1 showed a significant association with DII scores, the manuscript already acknowledges the study limitations and the need for further research. For this reason, the manuscript title has been retained.  While this study was not designed to establish reference intervals (typically requiring approximately 120 participants), the sample size of 101 participants is substantially larger than what would be expected for a pilot study and is appropriate for an exploratory, cross-sectional design assessing associations. |
| 2 | The introduction requires rewriting. It should be more in line with current knowledge and the stated purpose of the study. The concluding paragraph requires rewriting, and the stated purpose should be consistent throughout the manuscript—it should be concise and clearly define what the authors intend to demonstrate based on the data presented. Methodological elements are also unnecessary in this paragraph. | The final paragraph of the Introduction has been revised (see below) to clarify the study aim and remove methodological detail. The remainder of the Introduction was retained as it reflects current knowledge and provides the necessary context for the study.  *“The relationship between the inflammatory potential of maternal diet and the concentration of inflammatory markers in breast milk remains largely unexplored. This study aimed to investigate whether maternal dietary inflammatory status, assessed using the DII, is associated with concentrations of cytokines and chemokines in human breast milk in a cohort of Australian lactating women. Findings from this study aim to advance the understanding of the immunological profile of human breast milk and provide insight into the potential role of maternal diet in shaping immune-related components in mature human milk.”* |
| 3 | Methods:  Although the authors state that 'participants were screened against inclusion and exclusion criteria, as described in a previous study', I believe that a brief description of the study group, alongside a comparison to the general population of the country, should be included. I am concerned about using a 24-hour dietary recall to assess dietary intake and the Dietary Inflammatory Index (DII). Was this a single interview?  If so, a single 24-hour dietary recall does not accurately reflect habitual intake patterns and is therefore not suitable for demonstrating a relationship between diet and milk concentration.  Why was a single (morning only) collection of human milk used?  Milk sampling procedures call for repeated sampling over a period of at least four hours, ideally at the following times: 06:00–12:00, 12:00–18:00, 18:00–24:00 and 24:00–06:00.  Furthermore, was this a sufficient sample size (n=101 or n=99)? Was an attempt made to calculate the minimum sample size? | Participant eligibility criteria and recruitment procedures are described in the Methods, with additional cohort characteristics reported in the Results and original cohort publication. Briefly, participants were healthy lactating women breastfeeding singletons at 3–4 months postpartum, representing a relatively well-educated cohort. This level of detail has been included to improve clarity.  *“Dietary data was collected via the Australian ASA24 tool, which, while validated (21,22), relies on self-reporting and is therefore subject to recall bias and day-to-day variation in dietary intake. The ASA24 relies on participant recall and requires time to complete and a basic level of computer literacy. Dietary data were reviewed by an accredited practising dietitian to ensure completeness and plausibility, with predefined cut-offs applied to identify potential under- and over-reporting (<500 or >3500 kcal/day) (32). The 24-hour dietary recall may not fully represent habitual intake patterns (33). Additionally, a single morning milk sample was collected to standardise sampling conditions, minimise participant burden, and avoid disruption to infant feeding. As this study did not aim to assess diurnal variation, a single time-point sample was considered appropriate to meet the study objectives (34).”*  Dietary intake was assessed using the ASA24, a validated web-based dietary recall tool (20-22). As outlined in response to Reviewer 1, Comment 4, the 24-hour recall corresponding to the day prior to milk collection was used to align dietary intake with the paired milk sample. While a single recall may not fully represent habitual intake, participants were instructed to maintain their usual dietary patterns, and this limitation is acknowledged in the Discussion.  Breast milk samples were collected at a single time-point in the morning to standardise sampling conditions and minimise participant burden. Participants were instructed to collect samples between 07:00 and 11:00, having not been fed or expressed for at least two hours prior. While milk composition is known to vary across the day and over the course of lactation, a standardised single time-point sampling approach is commonly used in lactation research to assess associations with maternal characteristics.  A full 24-hour pooled collection was not feasible due to participant burden, study logistics, and potential impact on infant feeding, as repeated or prolonged expression may reduce milk availability for the infant. As this study did not aim to assess diurnal variation, a standardised single time-point sampling approach was considered appropriate to meet the study objectives.  The cohort is a convenience sample recruited over a defined period. Recruitment occurred over a defined period, resulting in 101 participants with dietary data and 99 samples available for laboratory analysis. The exploratory nature of the study and available recruitment window informed the final sample size. |
| 4 | Results:  Since inflammatory marker quantification was conducted on 99 participant breast milk samples (due to insufficient volume in the samples of two participants), why weren't all statistical analyses performed on n = 99? Table 1 contains data for n = 101. Why is this?  Table 2 cites 41 references (Global Daily Intake Mean (SD)[41]), but this item is not included in the bibliography (References) | Participant characteristics and dietary data were available for 101 participants and are therefore reported for the full cohort. Two milk samples lacked sufficient volume for quantification of inflammatory markers, leaving 99 samples available for laboratory analysis. Analyses involving milk cytokine concentrations were therefore conducted on 99 mothers, while cohort and dietary characteristics were reported for all 101 participants with available dietary data.  Dietary data for all participants were retained to fully characterise the cohort and avoid selectively excluding available data, with only paired dietary and milk data (n=99) used for analysis examining associations between diet and inflammatory markers.  The reference cited for the means of global dietary intake used in the DII calculation was incorrectly listed and has now been corrected. |
| 5 | Discussion  In my opinion, there is no need to recall the purpose of the study again.  The discussion should be expanded and strengthened with references to other literature — there is significantly more, and not all of them are included in the manuscript. Furthermore, since the authors state that no studies have been conducted on the link between maternal diet and inflammatory status or inflammatory markers in human breast milk, the discussion could focus on recommended diets, such as the Mediterranean diet, or the consumption of antioxidant compounds, such as carotenoids, polyphenols and antioxidant vitamins. | The purpose of the study is briefly restated at the beginning of the Discussion to orient the reader and frame interpretation of the findings, and this wording has been retained.  The Discussion focuses on the relationship between maternal DII scores and inflammatory markers in human milk. While broader dietary patterns such as the Mediterranean diet and antioxidant-rich foods are relevant, they were not examined in the current study, which specifically assessed dietary inflammatory potential using the DII. |
| 6 | The manuscript lacks a conclusion or a separate summary section. Implications for further research could also be separated.  In summary, it is important to emphasise that the issues addressed in this study are highly significant in terms of infant and general public health. | A separate Conclusion section has not been added because the final paragraph of the Discussion provides a concise summary of the key findings and their implications. Implications for future research are also addressed within the Discussion. |
